# Implementing Massive Parallel Sequencing into Biliary Samples Obtained through Endoscopic Retrograde Cholangiopancreatography for Diagnosing Malignant Bile Duct Strictures

**DOI:** 10.3390/ijms25179461

**Published:** 2024-08-30

**Authors:** Wonsuk Park, Jin Gwack, Joonhong Park

**Affiliations:** 1 Division of Gastroenterology, Department of Internal Medicine, Daejeon St. Mary’s Hospital, College of Medicine, The Catholic University of Korea, Seoul 06591, Republic of Korea; mdonekr@naver.com; 2 Department of Preventive Medicine, Jeonbuk National University Medical School, Jeonju 54907, Republic of Korea; gwackjin@jbnu.ac.kr; 3 Research Institute of Clinical Medicine of Jeonbuk National University-Biomedical Research Institute of Jeonbuk National University Hospital, Jeonju 54907, Republic of Korea; 4 Department of Laboratory Medicine, Jeonbuk National University Medical School and Hospital, Jeonju 54907, Republic of Korea

**Keywords:** extrahepatic cholangiocarcinoma, massive parallel sequencing, Oncomine Comprehensive Assay, Oncomine Pan-Cancer Cell-Free Assay, endoscopic retrograde cholangiopancreatography, biliary brush cytology, bile fluid

## Abstract

Despite advancements in radiologic, laboratory, and pathological evaluations, differentiating between benign and malignant bile duct strictures remains a diagnostic challenge. Recent developments in massive parallel sequencing (MPS) have introduced new opportunities for early cancer detection and management, but these techniques have not yet been rigorously applied to biliary samples. We prospectively evaluated the Oncomine Comprehensive Assay (OCA) and the Oncomine Pan-Cancer Cell-Free Assay (OPCCFA) using biliary brush cytology and bile fluid obtained via endoscopic retrograde cholangiopancreatography from patients with bile duct strictures. The diagnostic performance of MPS testing was assessed and compared to the pathological findings of biliary brush cytology and primary tissue. Mutations in *TP53*, *BRAF*, *CTNNB1*, *SMAD4*, and *K-/N-RAS* identified in biliary brush cytology samples were also detected in the corresponding bile fluid samples from patients with extrahepatic cholangiocarcinoma. These mutations were also identified in the bile fluid samples, but with variant allele frequencies lower than those in the corresponding biliary brush cytology samples. In control patients diagnosed with gallstones, neither the biliary brush cytology samples nor the bile fluid samples showed any pathogenic mutations classified as tier 1 or 2. Our study represents a prospective investigation into the role of MPS-based molecular testing in evaluating bile duct strictures. MPS-based molecular testing shows promise in identifying actionable genomic alterations, potentially enabling the stratification of patients for targeted chemotherapeutic treatments. Future research should focus on integrating OCA and OPCCFA testing, as well as similar MPS-based assays, into existing surveillance and management protocols for patients with bile duct strictures.

## 1. Introduction

Biliary tract cancers (BTCs), including extrahepatic cholangiocarcinoma (eCCA), ampullary carcinoma, and gallbladder cancer, are rapidly growing tumors with a high lethality rate [1]. The 5-year survival rate for patients with advanced or metastatic BTC is less than 10% [2]. Particularly, malignant strictures can be caused by carcinomas of the pancreatobiliary ducts, ampulla of Vater, liver, and, less frequently, metastatic cancers. Benign strictures may result from IgG4-related sclerosing cholangitis, primary sclerosing cholangitis (PSC), infections, iatrogenic injury, and other less common causes [3]. Diagnosing BTCs typically requires a tissue biopsy, but its invasiveness makes it impractical for routine use. Distinguishing between malignant and benign bile duct strictures is challenging and requires a multidisciplinary approach, including clinical examination, endoscopic procedures, radiographic imaging, biochemical testing (e.g., serum CA19-9), and pathological evaluation with supplementary studies. This differentiation is complicated by the fact that some benign strictures can predispose patients to malignancy [4]. For instance, PSC increases the risk of cholangiocarcinoma by 400 times compared to the general population. Endoscopic retrograde cholangiopancreatography (ERCP) is crucial in evaluating bile duct strictures by outlining the imaging features and extent of the disease [5]. However, fluoroscopic imaging during ERCP does not reveal definitive features that distinguish malignant from benign strictures. Additionally, direct cholangioscopic grading is unreliable and has poor interobserver consistency [6]. During ERCP, biliary brush cytology and forceps biopsies are used for pathological confirmation, but their sensitivity in detecting malignancy ranges from 8% to 67% [7,8,9]. To improve the detection of malignant strictures, adjunct techniques such as digital image analysis [10], fluorescence in situ hybridization (FISH) [11], and mutational testing for driver genes [12,13] have been developed, though their sensitivities vary between 14% and 60%. Given the low sensitivity of pathological evaluations and current supplementary studies, patients often undergo multiple ERCP procedures for diagnostic purposes, which can delay therapeutic decisions for malignant strictures by weeks or months, risking disease progression. Conversely, misdiagnosing a benign stricture as malignant can lead to unnecessary surgical resections, which carry significant morbidity and mortality rates [14]. Up to 15% of surgeries for suspected malignant strictures reveal benign disease, highlighting the need for better preoperative diagnostic methods [15].

To address this issue, less-invasive techniques are needed to assess tumor heterogeneity and the molecular changes in cancer cells. To date, noninvasive circulating tumor DNA (ctDNA) genotyping of plasma has become a cost-effective alternative to tissue biopsies in the diagnosis and management of many cancers [16,17,18]. Liquid biopsy can be useful for cancer detection, monitoring, and management. Fragmented DNA circulates in the cell-free component of whole fluids. The cell-free ctDNA in the blood is DNA released from apoptotic, circulating, or living tumor cells. ctDNA is about 140 nucleotides long and has a half-life of approximately 1.5 h. The analysis of ctDNA provides a noninvasive way to assess the genetic profiles of cancers in real time [19]. The potential role of ctDNA in the treatment of BTCs is particularly significant because endoscopic or percutaneous biopsy is invasive and often lacks accuracy. However, the diagnostic accuracy of ctDNA in blood and the concordance of results obtained from blood and tissue samples vary across many studies, limiting the diagnostic value of blood ctDNA for BTCs [20]. In cases of BTC, bile fluid directly contacts the tumor cells, and tumor-derived materials may be abundant in bile. Therefore, bile could be the ideal biofluid for exploring biomarkers and conducting molecular analysis of BTC [21].

Recent advancements have significantly improved our understanding of the genomic landscape of neoplasms in the bile duct system. Whole exome and genome sequencing studies have identified recurrent genomic alterations in several oncogenes and tumor suppressor genes, such as *CDKN2A*, *KRAS*, *TP53*, and *SMAD4* [22,23,24,25,26]. Some of these alterations, including *ATM* and *ERBB2*, may indicate susceptibility to specific anticancer therapies. Concurrently, novel molecular diagnostics have created new opportunities for studying preoperative samples [27]. Bile duct samples often contain small amounts of diagnostic material and heterogeneous cell populations, which can obscure or mimic malignancy. Therefore, a molecular assay must be highly sensitive to detect small proportions of mutated cells in these samples. Massively parallel sequencing (MPS) offers high analytical sensitivity and multigene analysis, making it an attractive option for assessing bile duct strictures [27,28,29]. In this study, we performed highly sensitive, targeted MPS testing, namely, Oncomine Comprehensive Assay (OCA) for biliary brush cytology and Oncomine Pan-Cancer Cell-Free Assay (OPCCFA) for bile fluid, targeting actionable genes commonly mutated, amplified, and/or deleted in bile duct malignancies. This test was conducted in a College of American Pathologists (CAP)-accredited clinical laboratory using biliary brush cytology and bile fluid obtained during ERCP. Instead of extracting DNA from cytological smears/slides, alcohol fixative (e.g., CytoLyt), or formalin-fixed paraffin-embedded (FFPE) tissue, which can reduce overall yields and quality, dedicated biliary brush cytology and/or bile fluid were submitted directly for targeted MPS testing after routine pathological evaluation. Our objectives were to prospectively evaluate targeted MPS testing on a small cohort of patients to (1) compare its performance as an adjunct to other diagnostic modalities, (2) determine its accuracy in detecting malignant strictures, and (3) assess the impact on patient management when genomic alterations are detected in bile duct samples.

## 2. Results

### 2.1. Clinicopathological Findings of the Study Population

For the primary analysis, biliary brush cytology samples were collected from 24 patients: 14 with suspected eCCA and 10 control subjects with gallstone (GS). Five eCCA samples were excluded due to poor DNA quality, which prevented the acquisition of molecular data. Detailed diagnostic and clinical follow-up information for each patient is provided in Table 1. During ERCP, nine patients were identified with indeterminate biliary strictures suggestive of eCCA. In six of these patients, the diagnosis of eCCA was confirmed by histology from either a repeated biopsy or resection material. In two patients, the diagnosis was based on a combination of radiologic imaging (computed tomography and magnetic resonance imaging) and ERCP with brush cytology indicative of malignant cells. The remaining patient was diagnosed with IgG4-related sclerosing cholangitis through histology, serology, and clinical follow-up. Biliary brush cytology samples from the control group were obtained from patients with biliary strictures that were later confirmed to be benign after at least 12 months of follow-up.

### 2.2. Mutation Analysis of Biliary Brush Cytology and Bile Fluid

Eight out of the 19 biliary brush cytology samples obtained from eCCA patients demonstrated one or more mutations based on targeted MPS testing, with a total of 21 mutations detected. Table 2 provides a comprehensive overview of the affected genes, nucleotide changes, relevant amino acid changes, and the allele molecular frequency for each mutation. Mutations were identified in the following genes: *TP53* (in six patients), *BRAF* (in three patients), *CTNNB1* (in two patients), *SMAD4* (in two patients), *RAS* (1 *KRAS* and 1 *NRAS* in two patients), *CDKN2A* (in one patient), *ERBB2* (in one patient), *FBXW7* (in one patient), *FGFR2* (in one patient), *MDM2* (in one patient), and *PIK3CA* (in one patient). Two different *RAS* mutations were found in two separate biliary brush cytology samples within the eCCA group. Notably, one eCCA sample (patient cc15) showed two different oncogenic *FBXW7* mutations, both present in transconfiguration in different DNA molecules, suggesting significant evolutionary pressure on the cancer to acquire mutations in this gene.

In contrast, only two biliary brush cytology samples from the control patients showed variants of uncertain significance, i.e., one *ATM* and one *TET2* variant (Table 3). Based on the cases with molecular data, the sensitivity of the targeted MPS testing was 100% (95% confidence interval [CI], 66.4% to 100%), and the specificity was 100% (95% CI, 71.5% to 100%). The odds ratio for a positive mutation analysis in the presence of eCCA was 119 (95% CI, 4.2 to 3311.3).

### 2.3. Mutations in Biliary Brush Cytology Compared with Bile Fluid Samples

**Figure 1:**
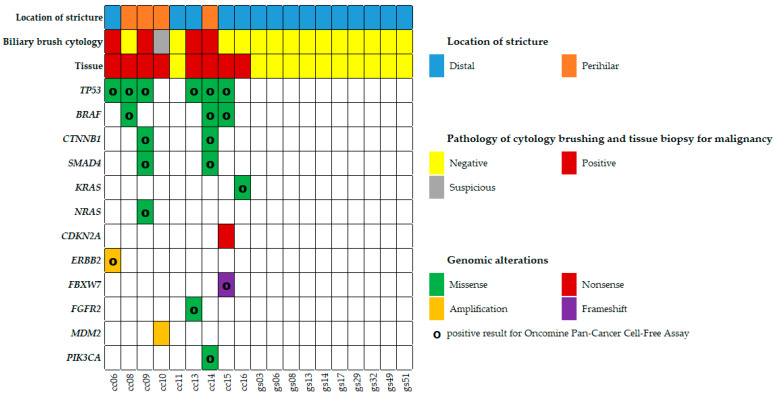
Correlative findings in patients with extrahepatic cholangiocarcinoma (eCCA) and confirmed gallstone (GS) as normal control. This figure presents correlative diagnostic findings in patients with eCCA and those with confirmed GS, analyzing results from pathological examination of biliary brush cytology, corresponding tissue pathology, and targeted massive parallel sequencing (MPS) testing. Targeted MPS testing identified individual genomic alterations associated with a high sensitivity (100%, 8/8) and specificity (100%, 11/11) for diagnosing adenocarcinoma involving the bile duct system (eCCA). The most frequently identified mutations detected by MPS testing, listed in decreasing prevalence, were *TP53*, *BRAF*, *CTNNB1*, *SMAD4*, and *KRAS/NRAS*, and other mutations classified as tier 1 or 2. The symbol “o” in genomic alterations indicates a positive result for Oncomine Pan-Cancer Cell-Free Assay.

Hotspot mutations covered by the targeted MPS test were identical in eight pairs of biliary brush cytology and bile juice samples (Figure 1). Recurrent mutations in *TP53*, *BRAF*, *CTNNB1*, *SMAD4*, and *K-/N-RAS* found in a biliary brush cytology sample were also detected in the corresponding bile juice sample. Interestingly, two of these bile juice samples exhibited *MDM2* and *CDKN2A* mutations with hotspots not included in the OPCCFA panel used in this study. These mutations were also identified in the bile fluid samples, but with variant allele frequencies lower than those in the corresponding biliary brush cytology samples. In control patients diagnosed with gallstones, neither the biliary brush cytology samples nor the bile fluid samples showed any pathogenic mutations classified as tier 1 or 2. Moreover, both MPS testing panels (OCA and OPCCFA) demonstrated higher sensitivity than biliary brush cytology alone (56%, 5/9), while maintaining high specificity. We have demonstrated that biliary brush cytology and liquid biopsy of bile, analyzed with MPS, can effectively detect DNA mutations.

## 3. Discussion

Despite advancements in radiologic, laboratory, and pathological evaluation, distinguishing between benign and malignant bile duct strictures remains a diagnostic challenge. Endoscopic or percutaneous tissue biopsy for the diagnosis of BTCs is invasive and has variable accuracy (50–90%) [30]. Consequently, tissue biopsy in BTC patients is challenging, and current guidelines do not recommend routine tissue biopsy for diagnosing BTC [31]. Biliary brush cytology is a well-established, safe, and widely used clinical method for diagnosing hepatobiliary strictures, often performed during ERCP procedures [32]. However, its diagnostic value has been questioned due to its low sensitivity, which typically ranges from 30% to 80% in previous studies [33,34,35]. This low sensitivity is attributed to the difficulty in detecting malignant tumor cells due to the limited number of cells collected during brush cytology. On the other hand, ctDNA, released into the bloodstream from dead tumor cells, offers several advantages over tissue biopsy in precision medicine, including sampling convenience and dynamic monitoring. However, the proportion of ctDNA in the blood is extremely low, necessitating highly sensitive methods to detect mutations, with allelic frequencies potentially as low as 0.1% [36]. In BTCs, bile fluid is in direct contact with tumor cells, making tumor-derived materials abundant in the bile. Additionally, BTCs exhibit heterogeneous features within a single mass, and oncological information from bile better reflects the tumor’s molecular characteristics than a tissue biopsy. Few studies have utilized bile ctDNA for the analysis or diagnosis of BTC. However, MPS has shown that mutated tumor DNA can be detected in bile with a high concordance rate compared to tumor tissue [37]. However, these studies included small patient cohorts, and the clinical significance of the mutant DNA remained unclear. Moreover, few studies have assessed the utility of bile fluid for ctDNA detection [38].

In the current study, we independently applied targeted MPS testing for evaluating bile duct strictures. Our study confirms the limitations of biliary brush cytology alone, which demonstrated a sensitivity of only 50% in diagnosing biliary tract malignancies among eight patients. However, it also introduces the promising solution of targeted MPS analysis. The targeted MPS testing demonstrated a sensitivity of 100% (95% confidence interval [CI], 66.4% to 100%) and a specificity of 100% (95% CI, 71.5% to 100%) in detecting mutations associated with eCCA in patients compared to controls. The odds ratio for a positive mutation analysis in the presence of eCCA was 119 (95% CI, 4.2 to 3311.3) compared to pathological evaluation alone for detecting malignant bile duct strictures involving the bile duct. Moreover, MPS testing (OCA and OPCCFA) demonstrated higher sensitivity than biliary brush cytology alone (56%, 5/9), while maintaining high specificity. We have demonstrated that biliary brush cytology and liquid biopsy of bile, analyzed with MPS, can detect DNA mutations. Genetic profiles of ctDNA in bile and plasma, as well as in tumor tissue, can be assessed. However, the detection rates of ctDNA in plasma were notably low in our study. Previous research has also reported varying detection rates (20–75%) and concordance between tissue and plasma results (25–79%) [39]. In our study, MPS testing revealed that the expressions of many genes encoding oncogenic signaling proteins were similar in both biliary brush cytology samples and bile fluid from patients with BTCs. This suggests that bile accurately reflects the biological and oncological properties of the tumor tissue. Therefore, our findings indicate that liquid biopsy of bile can serve as a viable alternative to tissue biopsy for ctDNA analysis, particularly with driver gene mutations playing a prognostic role in BTC patients. Additionally, monitoring ctDNA in bile offers real-time insights into treatment response and may potentially guide therapeutic adjustments in the future. The detection rate of driver gene mutations was 20 out of 22 in bile fluid ctDNA, which was comparable to that of biliary brush cytology. Furthermore, combining biliary brush cytology with targeted MPS analysis significantly improved the diagnostic accuracy, achieving 100% sensitivity. This suggests that targeted MPS could be a valuable tool for diagnosing eCCA using biliary brush cytology samples. Harbhajanka et al. further support the potential of combining biliary cytology with targeted MPS analysis. Their study demonstrated that supplementing cytomorphologic analysis with the molecular profiling of post-cytocentrifuged samples significantly increased sensitivity for diagnosing biliary tract malignancies to 93% while maintaining 100% specificity [40]. Thus, bile emerges as a superior biofluid compared to blood for ctDNA analysis in BTC patients. This study demonstrates, for the first time, the superiority of bile biopsy over plasma biopsy in BTC patients. In the context of obstructive jaundice caused by BTCs, exposure of the biliary epithelium and cancer cells to bile can lead to a high concentration of ctDNA derived from tumor tissue. However, our study did not use fresh plasma for comparison, and the use of frozen plasma may have adversely affected our results. Since no other study has compared bile and plasma for ctDNA detection, further comparative research is warranted [41].

To date, several studies have explored the application of targeted MPS testing in the context of bile duct strictures [11,27,28,29,42]. For instance, there was a retrospective cohort of 16 patients initially diagnosed with indeterminate bile duct biopsies that later progressed to cholangiocarcinoma [43]. Notably, the authors did not assess biliary brushing samples; instead, DNA for MPS was extracted from FFPE tissue blocks. Acknowledging the challenges posed by the low input and quality of DNA from FFPE tissue, they employed DNA enrichment techniques for analysis. While their study lacked a benign control group for comparison, the assay showed a sensitivity of 81%. Dudley et al. conducted a more extensive evaluation of MPS testing on bile duct samples. Their study included 73 bile duct and eight main pancreatic duct brushing samples [11]. In contrast to Bankov et al. [43], Dudley et al. [11] did not include bile duct biopsies in their cohort. They extracted DNA from CytoLyt-preserved samples, noting potential DNA degradation due to alcohol fixation, resulting in the failure of MPS testing in 11% of the brushing samples. Among the 65 remaining biliary brushing samples, Dudley et al. reported a sensitivity of 68% and a specificity of 97%, surpassing pathological evaluation [11]. However, they identified one false positive result, where the duration of follow-up after MPS testing was unclear, and the potential for dysplasia within the bile duct system could not be ruled out according to the authors. Besides MPS testing, several adjunctive techniques have been developed clinically to evaluate bile duct strictures, including droplet digital PCR analysis [10], single gene profiling [44], and multifocal FISH [45] for chromosomal polysomy. Among these ancillary studies, multicolor FISH has been widely adopted by many academic institutions for over a decade. Multi-color FISH relies on the observation that dysplastic and malignant neoplasms involving the bile duct often exhibit high frequencies of numerical chromosomal abnormalities. However, multi-color FISH typically achieves sensitivities of only 35–60%, with minimal technological improvement in recent years [46,47]. This technique is expensive, labor-intensive, prone to subjective interpretation errors, and requires significant technical expertise. Interpretation of multi-color FISH results necessitates an experienced pathologist to ensure accurate evaluation of epithelial cells for chromosomal abnormalities alongside morphological assessment. While a formal comparison between MPS testing and multi-color FISH was not conducted in this study, targeted MPS testing had higher sensitivity than multi-color FISH [11]. This superiority is not surprising given that MPS testing detects copy number alterations and single nucleotide variants across multiple genes. Moreover, with decreasing costs and the ability to batch multiple samples in a single run, MPS testing has become widely accessible to both academic and non-academic institutions. Therefore, MPS testing may be a more preferable option than multi-color FISH in the evaluation of bile duct samples. Demonstrating the clinical feasibility of MPS testing for ERCP-obtained biliary samples also sets the stage for broader applications of MPS testing to non-invasive sample types, such as bile fluid and serum.

In matched pair tumor and liquid biopsies, Astier et al. [48] observed an increased frequency of alterations in genes involved in genome integrity or chromatin remodeling, such as *ARID1A* (15%), *PBRM1* (9%), and *BAP1* (14%). There was variable concordance between ctDNA and tumor DNA. While there was a significant correlation in the total number of detected variants, there was some heterogeneity in the detection of actionable mutations. With a focus on clinically actionable alterations, all *IDH1* and *ATM* mutations were consistent in both tumor and liquid biopsies. Interestingly, eight out of nine *FGFR2* actionable alterations were exclusively detected in liquid biopsies. This observation may be attributed to the emergence of polyclonal secondary mutations following *FGFR2*-inhibitor therapy [49], as four out of five patients with these alterations had received pemigatinib or futibatinib and showed disease progression at the time of biopsy. Higher numbers of ctDNA alterations were observed in heavily pre-treated patients, which may reflect an increased genetic complexity of tumors over time and exposure to treatments. On the other hand, Nagai et al. demonstrated that cfDNA can be extracted from pancreatic and bile fluids of patients with pancreatic/bile duct strictures for genetic analysis [50]. They found that cfDNA analysis of pancreatic fluid and bile showed higher sensitivity for detecting malignancies compared to cytological assessments: pancreatic fluid (33% vs. 0%) and bile (53% vs. 19%). Moreover, the concentration of cfDNA in bile was higher than that in pancreatic fluid within the study cohort. This difference may be influenced by the more advanced tumor stages observed in the bile group compared to the pancreatic fluid group. These results suggest that cfDNA analysis of pancreatic fluid and bile, in conjunction with cytology, can serve as a valuable and complementary diagnostic tool. Interestingly, they showed that cfDNA fragments from pancreatic fluid and bile were much longer, consistent with other reports. This difference in fragment length could be attributed to variations in the type and activity of DNA restriction endonucleases present in these fluids. The longer cfDNA fragments observed in pancreatic fluid and bile are advantageous for high-sensitivity detection of gene alterations, particularly with newer DNA sequencing technologies that utilize long-read sequencing methods. In our study, the high concordance between mutations detected in biliary brush cytology and matched bile juice samples highlights the potential of bile juice as an alternative source for MPS analysis. This could provide a less invasive sampling method, particularly beneficial for patients who may not tolerate or require endoscopic procedures. However, the detection of *MDM2* and *CDKN2A* mutations in bile juice samples, which were not covered by the targeted MPS assay, highlights the need for a more comprehensive panel that includes additional target driver genes. Future studies could focus on developing broader MPS panels to capture a wider range of mutations potentially associated with eCCA.

Our study faced several limitations. The relatively small sample size was insufficient for robust statistical validation, which necessitates larger studies with more participants to confirm the diagnostic accuracy and generalizability of the MPS assay for eCCA. Therefore, our findings require verification with a larger patient cohort. This study focused primarily on hotspot mutations, and future research should investigate the role of other genetic alterations in eCCA development and their potential diagnostic and prognostic values. Additionally, exploring the potential of bile fluid as a non-invasive diagnostic tool for eCCA and developing more comprehensive MPS panels to detect a broader range of mutations associated with the disease are important areas for future investigation. MPS testing was not performed on tissue biopsy samples, making it challenging to assess the concordance of genomic alterations among primary tissue, biliary brush cytology samples, and bile fluid samples from each patient. For example, the result of MPS testing with OCA was positive for the biliary brush cytology samples, but it could not be tested using bile fluid because the targeted genes such as *MDM2* and *CDKN2A* were not included in the OPCCFA reagent, which is another limitation. Additionally, acquiring bile fluid through invasive procedures like ERCP or percutaneous transhepatic cholangiography is necessary. However, in cases of obstructive jaundice due to BTC or benign biliary strictures, biliary decompression is often essential. Given the ongoing clinical challenge of distinguishing BTCs from benign biliary strictures, liquid biopsy using bile could potentially offer clinical utility. This suggests that there may be aspects not explored in this study, and further investigations with larger sample sizes would be advantageous.

## 4. Materials and Methods

### 4.1. Sample Collection

Bile duct samples were prospectively collected from 24 patients in the Department of Internal Medicine, Daejeon St. Mary’s Hospital (Daejeon, Republic of Korea), between August 2017 and July 2018. These patients presented with bile duct strictures, of which nine were diagnosed with eCCA, and ten were diagnosed as benign controls with GS. The samples were obtained during standard ERCP procedures and included biliary brush cytology, forceps biopsies, or both. Biliary brush cytology samples were collected using multiple to-and-fro motions at the stricture site and placed in 15 mL of ThinPrep CytoLyt solution (Hologic, Marlborough, MA, USA) for processing within 24 h. All bile duct samples underwent routine cytopathological and pathological evaluation at the Department of Pathology, Daejeon St. Mary’s Hospital, within 24–48 h of ERCP. A separate biliary brush cytology sample was used for Oncomine Comprehensive Assay v3 (OCA v3, Thermo Fisher Scientific, Waltham, MA, USA), placed in a collection vial with DNA lysis buffer. Additionally, bile juice was collected in Cell-Free DNA BCT (Streck, Omaha, NE, USA) and used for Oncomine Pan-Cancer Cell-Free Assay (OPCCFA, Thermo Fisher Scientific). These samples were transported to the CAP-accredited clinical GC Genome laboratory (Yongin, Republic of Korea) within 24 h for processing. Forceps biopsies were processed similarly, with separate samples submitted for pathological examination and molecular profiling with OCA and OPCCFA. No minimum cellularity was required for testing. Patient demographics, clinical presentation, ERCP findings, and pathological diagnoses of biliary brush cytology and/or biopsies were recorded from medical records. A clinical diagnosis of malignancy was based on radiographic evidence of biliary stricture without acute cholangiopathy, clinical or radiographic progression after at least 12 months or death attributed to bile duct malignancy. Surgical resection and biopsy samples were diagnosed according to the World Health Organization (WHO) Classification of Tumours of the Digestive System criteria.

### 4.2. Massively Parallel Sequencing with Oncomine Comprehensive Assay

MPS was conducted prospectively within the clinical care framework, ensuring results were available within a 28-day turnaround in the CAP-accredited clinical GC Genome laboratory. Genomic DNA (gDNA) from biliary brush cytology samples was isolated using the RecoverAll Total Nucleic Acid Isolation kit (Thermo Fisher Scientific) following the manufacturer’s protocol. The extracted DNA was quantified using a Qubit 2.0 Fluorometer with the dsDNA HS Assay Kit (Thermo Fisher Scientific) and assessed for quantity and quality using the 2200 TapeStation (Agilent Technologies, Santa Clara, CA, USA). Targeted amplification-based MPS utilized primers targeting specific genomic regions of interest, as previously described [42]. Amplicons were barcoded, purified, and ligated with adapters tailored for subsequent sequencing on an Ion S5 XL sequencer (Thermo Fisher Scientific), in accordance with manufacturer guidelines. Data analysis employed Torrent Suite Software V.3.4.2 to detect point mutations, small insertions/deletions, and copy number alterations. The assay’s limit of detection was set at a mutant allele frequency (AF) of 3%, with a minimum coverage depth requirement of 500×. Each mutation detected was quantified by calculating its AF, derived from the ratio of reads of the mutant allele to the wild-type allele, expressed as a percentage [27]. Copy number variation (CNV) analysis, as previously outlined [51], defined gene amplification as the presence of ≥6 copies of a variant, validated using FISH analysis [28].

### 4.3. Massively Parallel Sequencing with Oncomine Pan-Cancer Cell-Free Assay

cfDNA extraction from bile fluid samples was conducted using the MagMax cfDNA Isolation Kit (Thermo Fisher Scientific) following the manufacturer’s protocol. Quantification of extracted DNA was performed on a Qubit 2.0 Fluorometer using the dsDNA HS Assay Kit (Thermo Fisher Scientific), and further quality assessment was conducted using the 2200 TapeStation (Agilent Technologies). Amplification-based targeted MPS was carried out with primers designed for specific genomic regions of interest, as previously described [52]. Amplicons were barcoded, purified, and ligated with specific adapters. A minimum of 1.3 ng of ctDNA was utilized for library preparation. Libraries were quantified using the TapeStation, and templating was performed with the Ion 540 Kit-Chef (Thermo Fisher Scientific). Samples underwent sequencing at high depth on the Ion S5 XL sequencer, and analysis was conducted using Torrent Suite Software v.5.2, Ion Reporter versions 5.6 and 5.10 (Thermo Fisher Scientific), with Human Genome Build 19 as the reference. The Oncomine TagSeq Pan-Cancer Liquid Biopsy w2.1 workflow was employed with default parameters, utilizing Oncomine variant annotator version 2.4 for variant annotation. The mean sequencing depth across all samples was 21,358×. Copy number variation (CNV) sensitivity was set to medium, defining a CNV alteration with a cut-off value of 2.0-fold or higher. Single nucleotide variant (SNV) detection sensitivity was set at 0.3%, with a recommended DNA input of 20 ng to achieve a 0.1% SNV limit of detection. The range of cfDNA input for library preparation varied from 1.3 to 20 ng, adhering strictly to the manufacturer’s instructions for library preparation and sequencing protocols.

### 4.4. Classification of Genomic Alterations

Variant interpretation adhered to the 2017 Association for Molecular Pathology (AMP)/American Society of Clinical Oncology (ASCO)/College of American Pathologists (CAP) joint consensus guidelines, employing a tier-based system [53]. Only Tier I and Tier II variants were considered for further analysis; their presence constituted a positive result, whereas the absence of these variants indicated a negative result. Clinically relevant genomic alterations refer to mutations that can be targeted by existing anticancer drugs on the market or those being investigated in clinical trials. Specifically, genomic alterations potentially targetable with reported kinase inhibitors, assuming wild-type *KRAS, NRAS*, and *HRAS* genomic profiles, include *ALK*, *BRAF*, *EGFR*, *ERBB2*, *FGFR1*, *FGFR2*, *FGFR3*, and *MET* [54,55,56,57].

### 4.5. Statistical Analysis

Statistical comparisons of mutational statuses were assessed using Fisher’s exact test for dichotomous variables. Receiver operating characteristic (ROC) curves were generated for the training cohort to evaluate biomarker performance and establish optimal cut-off levels using the Youden index. Final cut-offs were selected to maximize the area under the curve (AUC) calculated by the trapezoidal method, considering both biological rationale and model simplicity. Sensitivity and specificity were computed using standard 2 × 2 contingency tables for cases with confirmed diagnostic pathology. Repeat testing was not employed unless otherwise specified to calculate sensitivity and specificity for individual biomarkers. All statistical analyses were conducted using MedCalc statistical software version 19.8.3 (MedCalc Software, Ltd., Ostend, Belgium), with statistical significance defined as a *p*-value of less than 0.05.

## 5. Conclusions

Our study represents a prospective investigation into the role of MPS-based molecular testing in evaluating bile duct strictures. Our findings underscore the clinical benefit of complementing the standard pathological evaluation of bile duct samples with OCA in biliary brush cytology and OPCCFA in bile juice. Particularly, ctDNA is abundant in bile samples, and somatic variants of oncogenes in bile ctDNA can be detected using MPS. The high mutational concordance among biliary brush cytology, bile juice, and tumor tissue indicates that liquid biopsy to extract bile ctDNA can be effectively used to detect somatic variants of driver genes for the diagnosis and prognosis of BTCs. Utilizing bile biopsy may lead to more rational, personalized, and targeted therapeutic approaches for BTCs in the future. Moreover, MPS-based molecular testing shows promise in identifying actionable genomic alterations, enabling the stratification of patients for targeted chemotherapeutic treatments. Future research efforts should focus on integrating OCA and OPCCFA testing and similar MPS-based assays into existing surveillance and management protocols for patients with bile duct strictures.

## Figures and Tables

**Table 1:** Clinicopathological findings, treatment, and prognosis for nine patients initially suspected of extrahepatic cholangiocarcinoma.

Case	Diagnosis		Pathology	Cytology	Treatment	Prognosis	OS (Day)
	Initial	Confirmed					
cc06	eCCA	dBDC	AC	Positive *	PPPD, Adjuvant ChT	death	622
cc08	eCCA	pCCA	AC	Negative ^†^	ConserTx	death	239
dc09	eCCA	pCCA	AC	Positive	ConserTx	F/U loss	325
cc10	eCCA	pCCA	AC	Suspicious ^‡^	ConserTx	F/U loss	120
cc11	eCCA	SC	SC, IgG4-related	Negative	ConserTx	alive	2284
cc13	eCCA	dBDC	AC	Positive	ConserTx	death	267
cc14	eCCA	pCCA	AC	Positive	PPPD	F/U loss	408
cc15	eCCA	dBDC	AC	Negative	PPPD	alive	2001
cc16	eCCA	dBDC	AC with SRC	Negative	ConserTx	death	303

eCCA, extrahepatic cholangiocarcinoma; dBDC, distal bile duct cancer; pCCA, perihilar cholangiocarcinoma; SC, sclerosing cholangitis; AC, adenocarcinoma; SRC, signet ring cell; PPPD, pylorus preserving pancreatoduodectomy; ChT, chemotherapy; ConserTx, conservative treatment; F/U, follow up. * Positive, this indicates the presence of malignant cells, confirming cancer; ^†^ Negative, no malignant cells were detected. This does not necessarily rule out cancer, but further investigation may be needed. ^‡^ Suspicious, the cells show features suggestive of malignancy but are not conclusive.

**Table 2:** Massive parallel sequencing analysis results using Oncomine comprehensive assay v3 for biliary brush cytology and Oncomine pan-cancer cell-free assay for bile juice in nine patients initially suspected of extrahepatic cholangiocarcinoma.

Case	S/A (y)	Gene	Mutation	Mutation Change	Depth	VAF	rsID	Class	OPCCFA
			Type		(×)	(%)			(%)
cc06	M/61	*TP53*	missense	c.832C>G/p.P278A	1965	64	rs17849781	tier 2	3.7
		*ERBB2*	amplification (8 copies)					tier 2	1.8
cc08	M/72	*TP53*	missense	c.1039G>A/p.A347T	2000	46	rs1597349147	tier 2	3.7
		*BRAF*	missense	c.1397G>A/p.G466E	1999	6	rs121913351	tier 2	0.8
cc09	F/79	*TP53*	missense	c.535C>T/p.H179Y	1694	70	rs587780070	tier 2	3.1
		*CTNNB1*	missense	c.134C>G/p.S45C	2000	14	rs121913409	tier 2	0.6
		*SMAD4*	missense	c.1051G>A/p.D351N	2000	15	rs1057519739	tier 2	0.3
		*NRAS*	missense	c.182A>T/p.Q61L	2000	43	rs11554290	tier 2	2.6
cc10	M/58	*MDM2*	amplification (6 copies)					tier 2	N.A.
cc11	M/64	Not Detected							N.D.
cc13	M/76	*TP53*	missense	c.707A>G/p.236C	1970	62	rs730882026	tier 2	2.9
		*FGFR2*	missense	c.1144T>C/p.C382R	1885	36	rs121913474	tier 1	1.4
cc14	F/76	*TP53*	missense	c.523C>T/p.R175C	1222	45	rs138729528	tier 2	3.9
		*BRAF*	missense	c.1781A>G/p.D594G	1022	28	rs121913338	tier 2	2.6
		*CTNNB1*	missense	c.134C>G/p.S45C	1999	67	rs121913409	tier 2	2.5
		*SMAD4*	missense	c.1081C>T/p.R361C	2000	30	rs80338963	tier 2	0.7
		*PIK3CA*	missense	c.1624G>A/p.E542K	1963	37	rs121913273	tier 2	0.3
cc15	M/71	*TP53*	missense	c.817C>T/p.R273C	2000	31	rs121913343	tier 2	3.4
		*BRAF*	missense	c.1780G>A/p.D594N	2000	16	rs397516896	tier 2	0.3
		*CDKN2A*	nonsense	c.205G>T/p.E69*	1434	47	rs121913383	tier 2	N.A.
		*FBXW7*	frameshift	c.58delA/p.R20Efs*9	1981	15	N.A.	tier 2	2.2
		*FBXW7*	frameshift	c.1218delG/p.W406Cfs*9	1994	13	N.A.	tier 2	3.1
cc16	F/90	*KRAS*	missense	c.182A>T/p.Q61L	2000	26	rs121913240	tier 2	4.3

S/A (y), sex/age (year); VAF, variant allele frequency; rsID, reference SNP cluster ID; OPCCFA, Oncomine Pan-Cancer Cell-Free Assay; N.A., not available; N.D., not detected.

**Table 3:** Massive parallel sequencing analysis results using Oncomine comprehensive assay v3 for biliary brush cytology and Oncomine pan-cancer cell-free assay for bile in ten patients with gallstones.

Case	S/A (y)	Gene	Mutation	Mutation Change	Depth	VAF	rsID	Class	OPCCFA
			Type		(×)	(%)			
gs03	F/34	Not Detected							N.D.
gs06	F/62	*TET2*	missense	c.4076G>A/p.R1359H	1999	29	rs775677220	tier 3	N.A.
gs08	M/57	Not Detected							N.D.
gs13	F/59	Not Detected							N.D.
gs14	F/64	Not Detected							N.D.
gs17	M/47	Not Detected							N.D.
gs29	M/55	*ATM*	missense	c.7328G>A/p.R2443	2000	13	rs587782310	tier 3	N.A.
gs32	M/42	Not Detected							N.D.
gs49	F/43	Not Detected							N.D.
gs51	M/56	Not Detected							N.D.

S/A (y), sex/age (year); VAF, variant allele frequency; rsID, reference SNP cluster ID; OPCCFA, Oncomine Pan-Cancer Cell-Free Assay; N.A., not available; N.D., not detected.

## Data Availability

Data are contained within the article.
